# New mutations in BBS genes in small consanguineous families with Bardet-Biedl syndrome: Detection of candidate regions by homozygosity mapping

**Published:** 2010-02-01

**Authors:** Ines Pereiro, Diana Valverde, Teresa Piñeiro-Gallego, Montserrat Baiget, Salud Borrego, Carmen Ayuso, Charles Searby, Darryl Nishimura

**Affiliations:** 1 Facultad de Biología, Universidad de Vigo, Spain; 2 Hospital de Sant Pau, Barcelona, Spain; 3 Hospital Virgen del Rocio, Sevilla, Spain; 4 Fundación Jiménez Díaz, Madrid, Spain; 5 Howard Hughes Medical Institute, University of Iowa, Iowa City, IA; 6 Department of Pediatrics, University of Iowa, Iowa City, IA

## Abstract

**Purpose:**

Bardet-Biedl syndrome (BBS, OMIM 209900) is a rare multi-organ disorder in which BBS patients manifest a variable phenotype that includes retinal dystrophy, polydactyly, mental delay, obesity, and also reproductive tract and renal abnormalities. Mutations in 14 genes (*BBS1–BBS14*) are found in 70% of the patients, indicating that additional mutations in known and new BBS genes remain to be identified. Therefore, the molecular diagnosis of this complex disorder is a challenging task.

**Methods:**

In this study we show the use of the genome-wide homozygosity mapping strategy in the mutation detection of nine Caucasian BBS families, eight of them consanguineous and one from the same geographic area with no proven consanguinity.

**Results:**

We identified the disease-causing mutation in six of the families studied, five of which had novel sequence variants in *BBS3*, *BBS6,* and *BBS12*. This is the first null mutation reported in *BBS3*. Furthermore, this approach defined homozygous candidate regions that could harbor potential candidate genes for BBS in three of the families.

**Conclusions:**

These findings further underline the importance of homozygosity mapping as a useful technology for diagnosis in small consanguineous families with a complex disease like BBS.

## Introduction

Bardet-Biedl syndrome (BBS, OMIM 209900) is a pleiotropic human genetic disorder that has as primary phenotypic features early-onset retinitis pigmentosa, obesity, dystrophic extremities, and polydactyly together with reproductive tract and renal abnormalities and a variable degree of cognitive impairment [1].

BBS is rare in the general population, with prevalence rates in North America and Europe ranging from 1:140,000 to 1:160,000 live births [2,3]. However, a higher incidence has recently been reported in certain isolated populations, such as those in Newfoundland, Kuwait, and the Faroe Islands [4–6].

BBS has a heterogeneous genetic nature with at least 14 genes (*BBS1–BBS14*) identified to date [7,8]; this shows considerable interfamilial and intrafamilial variation in the phenotype [7]. BBS has been defined as an autosomal disorder, but some families harbor a model of complex inheritance where additional mutations in other genes modify the phenotype [7,8]. Mutation screening of the known BBS genes has resulted in the identification of the causative mutations in approximately 70% of the BBS families, indicating that additional BBS genes remain to be identified [9].

BBS is a difficult disease upon which to perform molecular diagnostic studies. It is tedious and time consuming to search for mutations in each of the 14 genes described as some of them have been implicated in only a few cases and several genes present large coding regions in which it is necessary to search for mutations.

The genome-wide homozygosity mapping approach has been useful for molecular diagnosis studies of complex diseases, for the search for new mutations in known genes, as well as for identifying new disease genes [10–13]. This technology is based on the assumption that patients with a recessive disease and born from a consanguineous union are likely to be homozygous for the disease-causing mutation and for polymorphisms in the region surrounding this mutation [14].

Therefore, the aim of this study was to use genome-wide homozygosity mapping to diagnose BBS patients from consanguineous families. Identifying homozygous chromosomal regions in these families allowed us to search for mutations in the known BBS genes and to find new BBS loci within candidate regions.

## Methods

**Figure 1:**
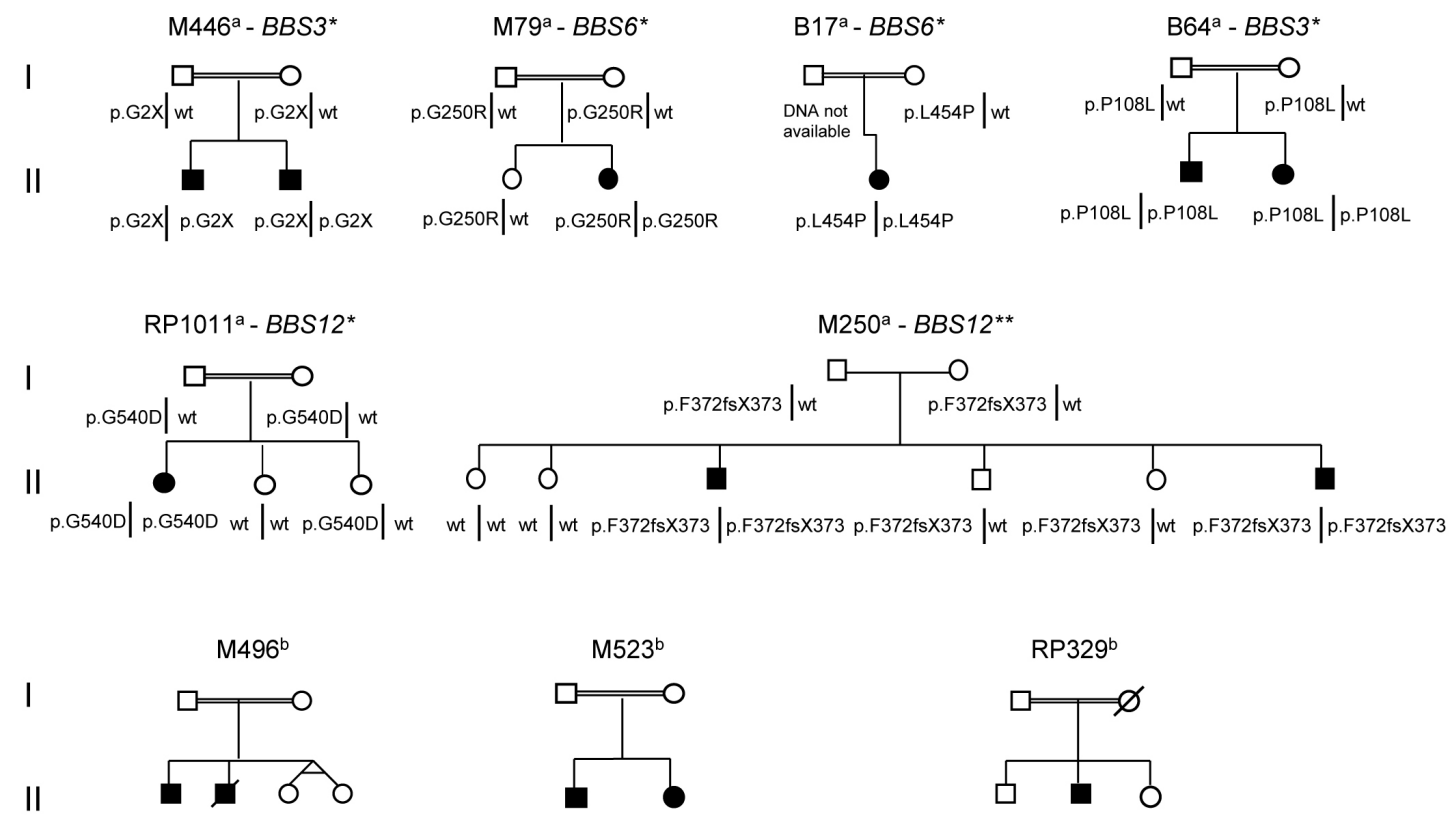
Pedigrees of the Bardet-Biedl syndrome families studied showing the segregation of the mutations found. Each pedigree were named with the family code and the mutated BBS gene. ^a^Families with mutations detected, ^b^families without mutations detected. *Novel mutations, **mutation reported by Stoetzel et al. [16] wt denotes wild type.

Nine Caucasian families from Spain with one or more affects of BBS, eight of them consanguineous and one (M250) from the same geographic area with no proven consanguinity, were selected from our pool of Spanish BBS patients. The pedigrees of the families and the gender of the BBS affects are shown in Figure 1. The recruitment of patients and relatives was performed through the Retinal Dystrophy Investigation Spanish Network (EsRetNet). Informed consent was obtained from all patients and relatives after the nature and possible consequences of the study had been explained.

Clinical diagnosis was based on the criteria described by Beales et al. [1], where the presence of at least three main and two minor phenotypic characteristics are necessary. Clinical information of the BBS patients is summarized in Table 1. No detailed clinical data were available for patients from the B64 family, as the clinical history was lost.

**Table 1:** Clinical characteristics of BBS patients studied.

**Patient**	**Retinitis pigmentosa**				**Polydactyly**	**Obesity**	**Mental delay**	**Other**
	**NB (onset)**	**Reduced visual acuity**	**Reduced visual field**	**Fundus and ERG**				
M446 II.1	5 yo	22 yo	yes	abolished	-	+/−	-	Dysmorphic facial features
M446 II.2	5 yo	CF	yes	abolished	+ (feet)	+	-	Hearing loss
M79 II.2	3 yo	6 years (20°)	ND	abolished (at 12 yo)	+	+	+	
B17 II.1	yes		ND	abolished	+ (left hand)	+	+	Renal insufficiency, arterial hypertension
M250 II.3	infancy	20 yo	ND	ND	+ (left foot)	+	+	Hypogonadism
M250 II.6	15 yo	20 yo	ND	ND	+	+	+	Hypogonadism, photophobia, myopia
RP1011 II.1	9 yo	9 yo	1/8 1/10	Spicular pigment scotomae	+	+	+/−	Psychomotor delay, photophobia
M496 II.1	20 yo	reduced	yes	ND	+	-	-	Hirschprung disease
M523 II.1	yes	reduced	yes	ND	+	+	+	
M523 II.2	yes	reduced	yes	ND	-	+	-	
RP329 II.2	yes	reduced	yes	ND	-	+	+	Photophobia, psychomotor and developmental delay

NB: night blindness, CF: counting fingers, yo: years old, ERG: electroretinogram, +/−: mild feature, ND: not determined.

DNA extraction from 10 ml of whole blood samples was performed by a salting-out procedure described by Miller et al. [15].

In all BBS patients, except those from the M496 family, as the DNAs were degraded, the presence of previously described mutations in the *BBS1–BBS10* genes were excluded by using a genotyping microarray based on arrayed primer extension (APEX) technology (Asper Ophthalmics, Tartu, Estonia).

DNA from the BBS patients was genotyped on an Affymetrix GeneChip Human Mapping 50K (Affymetrix, Santa Clara, CA) to detect homozygous regions, previously described by Nishimura et al. [13]. When a known BBS gene was localized in a homozygous region of a given family, the coding sequence and the consensus splice sites of that gene were sequenced. To exclude some of the homozygous regions detected in family RP329, we also genotyped the healthy siblings of this family with the same methodology, and this gave us information about the homozygous regions shared only by the BBS patients in this family.

To determine if novel BBS gene variants (p.G250R and p.L454P [*BBS6*] and p.P108L [*BBS12*]) were present in control samples, we sequenced 100 chromosomes of a control population provided by the Complejo Hospitalario Universitario de Vigo (Vigo, Spain). The *BBS12* p.G540D mutation was tested by a BsgI restriction assay. In the restriction assay a 665 bp amplicon was cleaved into three fragments of 434, 129, and 102 bp, respectively, when the normal allele is present; whereas the p.G540D mutation abolishes one BsgI site producing two fragments of 434 bp and 231 bp, respectively.

To evaluate the potential pathogenic effect of the missense mutations, we used a homolog protein alignment program. Furthermore, we used a splice site scoring program to test for DNA variants effect on splicing.

## Results

The homozygosity regions detected in seven of the nine families (M446, M79, B17, M250, B64, RP1011, and M496) included known BBS genes. The sequencing of these BBS genes revealed sequence variants in six families that were always detected as homozygous. In five families the detected sequence variants were novel (Figure 1).

In the M446 family, we identified a novel homozygous nonsense mutation p.G2X (c.4G>T) in *BBS3.* In families M79 and B17, two novel *BBS6* missense mutations were found, p.G250R (c.748G>A) and p.L454P (c.1361T>C), respectively. Finally, BBS12 missense mutations in homozygous state were detected in families B64, p.P108L (c.3232C>T) and RP1011, p.G540D (c.1620 G>A). Another *BBS12* mutation, a previously described frameshift mutation [16], p.F372fsX373 (c.1114delTT), was detected in both patients from family M250. In each family all of the mutations segregated with the disease (Figure 1), consistent with a pattern of autosomal recessive inheritance. Moreover, the mutations detected were not found in 100 ethnically matched control chromosomes. Determination of mutation segregation in families B17 and B64 was not possible.

**Figure 2:**
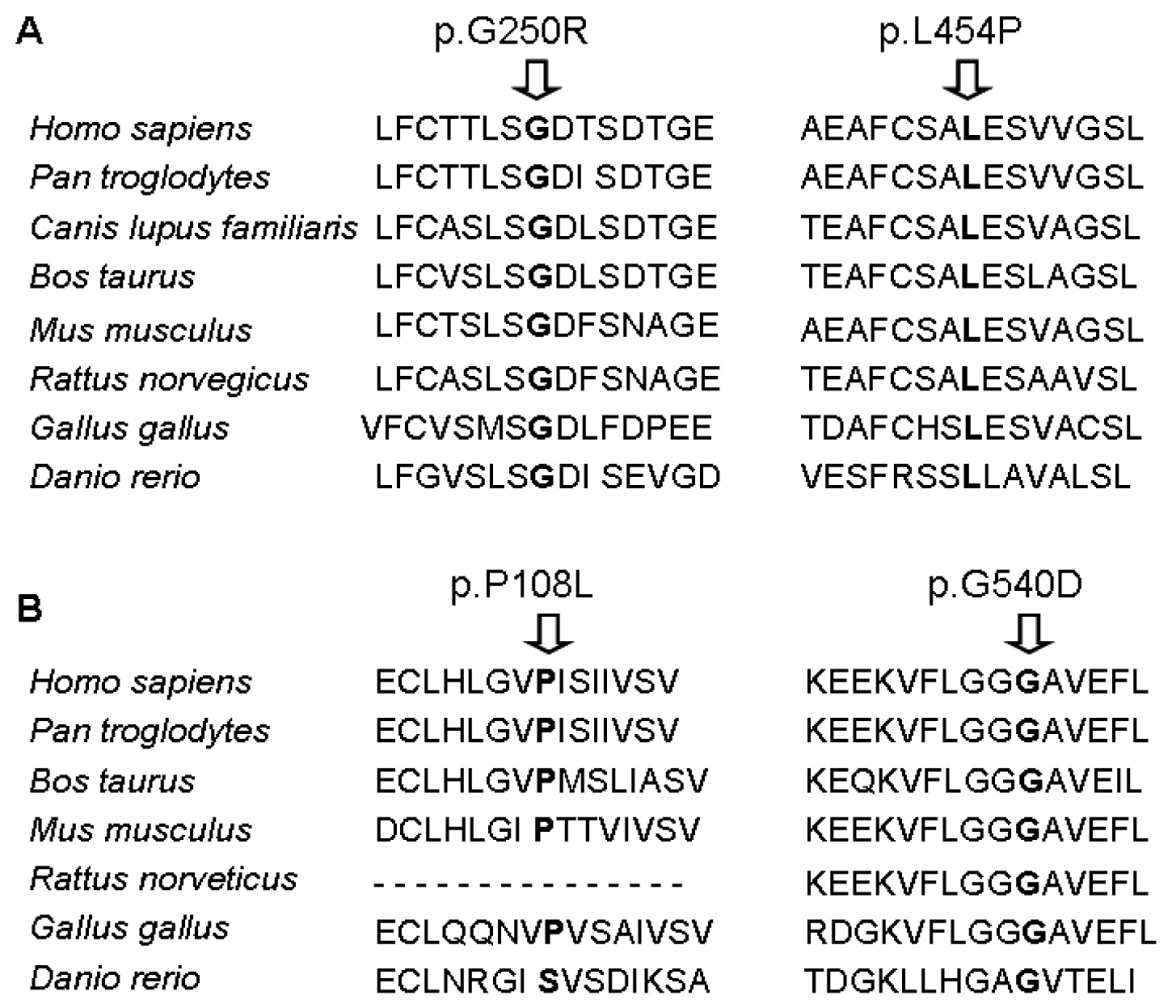
Sequence alignment of BBS6 protein and BBS12 protein of several species, showing the conservation of the amino acid implicated in the missense mutations. **A**: BBS6 mutations (p.G250R and p.L454P); **B**: BBS12 mutations (p.P108L and p.G540D).

The application of the splice site scoring programs showed that none of the changes detected seemed to modify the splice site or create new splice sites. Protein sequence alignment showed that all of the novel missense mutations are localized within conserved regions (Figure 2).

For family M496, homozygous regions included the *BBS3* and *BBS6* genes, but the sequencing of the exon and intronic boundaries of those genes did not reveal candidate pathogenic variants. However, for the M496, RP329, and M523 families, we were able to detect several homozygous regions along the genome (Table 2). In the case of family RP329, the candidate homozygous regions were redefined by the genotyping of the unaffected siblings.

**Table 2:** Homozygous regions identified by SNP genotyping in the BBS families without mutation detected.

**Family**	**Chromosomal region**	**Interval size (Mb)**
M496	3p12.3-q13.13*	32.46
	9p22.2-p13.3	18.69
	13p13-q12.13	25.06
	13q13.3-q21.31	25.72
	16q21-q24.3	25.72
	20p12.3-q13.12**	36.08
M523	1q23.3-q31.3	34.05
	6p25.3-p24.1	11.91
	7p14.2-q11.22	35.41
	9p24.1-p22.3	9.72
RP329	1p21.3-p13.3	13.70
	1q32.3-q41	8.61
	15q11.2-q22.31	45.66
	18q12.3-q21.2	6.96

* Homozygous region including *BBS3* gene. ** Homozygous region including *BBS6* gene.

## Discussion

We report the utility of high-density technologies, such as genome-wide Single Nucleotide Polymorphism (SNP) genotyping, in identifying disease-causing mutations in recessive complex diseases, such as the Bardet-Biedl syndrome. In this study, the homozygosity mapping of eight consanguineous families and one family from a small geographic area allowed us to identify in six families six sequence variants in known BBS genes.

Of the BBS gene sequence changes detected, one was previously described and five were novel variants (one nonsense mutation and four missense mutations). As evidence of the pathogenicity of the novel missense variants, (i) all mutations were found to localize within conserved regions of the genes, (ii) the mutations segregated with the disease, (iii) the mutations were absent in 100 control chromosomes, and (iv) the mutations were not found in the database SNP (dbSNP) database.

The *BBS3* (also known as ADP-ribosylation factor-like protein 6 [*ARL6*]) nonsense mutation (p.G2X) seems to be a null mutation because, in theory, it does not allow the formation of the BBS3 protein. Although this mutation may be disease-causing in the M446 family, its consequences do not seem to be any worse than in the other BBS families because there are no major phenotypic differences among the families studied (Table 1). However within the two affected brothers with the same mutation, the phenotype is clearly different. For the younger brother the progression of the disease has been quite slow, with a reduction of the visual field at the age of 18 and a diminished visual acuity at 22 and no polydactyly. For the proband in this family, major clinical characteristics have been observed: polydactyly, obesity, retinitis pigmentosa, dysmorphic facial features, and hearing loss. It was not possible to obtain an electroretinogram (ERG) from the proband. None of the two brothers shows mental delay. ARL6 is a member of a subgroup of the ARF family adenosine diphosphate ribosylation factor (ADP-ribosylation factor) that is involved in diverse cellular functions, including the regulation of intracellular traffic. It is a cytosolic protein with the highest levels of mRNA being present in the brain and kidney [17]. The *ARL6* mRNA expression pattern during early mouse embryonic development is on the ventral layer node, which is distinguished by the presence of a single, motile, central cilium [18]. Functional analyses of this mutation are needed to elucidate the role of this protein in the development of the clinical characteristics of BBS.

For families M79 and B17, the mutation was detected in the *BBS6* gene. The patients from the M79 family and the B17 family are homozygous for the p.G250R mutation and the p.L454P mutation, respectively. Both changes affect the Pfam domain of the protein by modifying the ATPase activity and the protein-binding site. For the p.G250R mutation, the neutral glycine with a simple lateral chain changed to the basic arginine with a longer lateral chain. For the p.L454P mutation, although the apolar characteristic of the amino acid did not change, the lateral chain of proline has special characteristics that could influence protein configuration.

The clinical ocular phenotype of patient M79 showed an onset of night blindness at the age of 3, with diminished visual acuity and a diminished peripheral visual field and an abolished ERG at the age of 12. Clinical characteristics typical of BBS are present, except for renal affectation. For patient B17, a typical visual phenotype with night blindness and an abolished ERG was observed, together with other typical clinical characteristics, such as renal insufficiency and high arterial hypertension.

For families M250, B64, and RP1011, mutations in the *BBS12* gene have been detected (p.F372fsX373, p.P108L, and G540D, respectively). For the two affected brothers of family M250, differences in the phenotype have been reported. One brother shows a more severe phenotype, with polydactyly in all extremities, photophobia, and myopia. The retinitis pigmentosa (RP) debuted with night blindness at the age of 15 with a decreased visual acuity at the age of 20. The other brother developed night blindness during infancy, a reduced visual acuity at the age of 20 and was born with polydactyly in the left foot. Both brothers had hypogonadism. For family RP1011, the age of onset of the RP is in the first decade of life, showing an aggressive progression as well as polydactyly in both hands and feet, obesity, and psychomotor delay.

The previously discussed phenotypic differences between affected siblings carrying the same BBS mutation could be explained by the presence of a third mutation. This third mutation could have a modifying effect modulating the expression of the disease phenotype [19].

The sequencing analysis performed to exclude the implication of known BBS genes did not allow us to rule out the presence of mutations in introns or promoter regions or to rule out small deletions. However, the use of high-density SNP genotyping for homozygosity mapping seems to be a good screening strategy in consanguineous families and families from a small geographic area to efficiently focus on mutation detection.

Furthermore, this approach defines homozygous candidate regions that could harbor potential candidate genes for BBS. In the families that did not harbor a mutation in known BBS genes, the candidate regions did not overlap. This suggests that the disease in each family might be caused by different genes. Nevertheless, the number of families included in this study was small, so the candidate regions identified were large and numerous and will thus include many false positives [20]. The delimitation of these regions using unaffected siblings was limited; it was only possible in one BBS family (RP329). Therefore, additional studies, such as in silico analyses or higher resolution SNP analysis, are necessary to refine such regions.

This finding further underlines the importance of homozygosity mapping as a tool for identifying genes and mutations involved in recessively inherited diseases. This strategy has been performed in BBS consanguineous families to detect new genes, such as *BBS9* [13], *BBS11* [11], and *BBS12* [16]; in other retinal pathologies, such as Leber congenital amaurosis, this strategy allowed the identification of novel mutations in the *LCA5* gene [21].
